# Risk-taking, peer-influence and child maltreatment: a neurocognitive investigation

**DOI:** 10.1093/scan/nsx124

**Published:** 2017-10-23

**Authors:** Ferdinand Hoffmann, Vanessa B Puetz, Essi Viding, Arjun Sethi, Amy Palmer, Eamon J McCrory

**Affiliations:** 1 Division of Psychology and Language Sciences, University College London, London, UK,; 2 Anna Freud National Centre for Children and Families, London, UK

**Keywords:** childhood maltreatment, risk-taking, peer influence, fMRI

## Abstract

Maltreatment is associated with increased risk of a range of psychiatric disorders, many of which are characterized by altered risk-taking propensity. Currently, little is known about the neural correlates of risk-taking in children exposed to maltreatment, nor whether their risk-taking is atypically modulated by peer influence. Seventy-five 10- to 14-year-old children [maltreated (MT) group: *N* = 41; non-maltreated Group (NMT): *N* = 34] performed a Balloon Analogue Risk Task (BART), under three different peer influence conditions: while alone, while being observed by a peer and while being encouraged by a peer to take risks. The MT group engaged in less risk-taking irrespective of peer influence. There was no differential effect of peer influence on risk-taking behaviour across groups. At the neural level, the right anterior insula (rAI) exhibited altered risk sensitivity across conditions in the MT group. Across groups and conditions, rAI risk sensitivity was negatively associated with risk-taking and within the MT group greater rAI risk sensitivity was related to more anxiety symptoms. These findings suggest that children with a history of maltreatment show reduced risk-taking but typical responses to peer influence. Abnormal rAI functioning contributes to the pattern of reduced risk-taking and may predispose children exposed to maltreatment to develop future psychopathology.

## Introduction

Childhood maltreatment is associated with significantly increased risk of a range of psychiatric disorders (Gilbert *et al.*, 2009) as well as poor economic productivity across the lifespan (Currie and Widom, 2010). However, relatively little is known about the neurocognitive mechanisms that may underpin these associations. According to the theory of latent vulnerability, maltreatment results in measurable alterations in a number of neurocognitive systems that reflect calibration to neglectful and/or abusive early environments (McCrory and Viding, 2015; McCrory *et al.*, 2017). A general principle of the theory is that these changes may represent (at least in part) an adaptation in response to an adverse caregiving environment during childhood. However, such alterations are equally thought to incur a longer term cost as they may mean that the individual is poorly optimized to negotiate the demands of other, more normative environments and be vulnerable to future stressors (McCrory and Viding, 2015).

To date, the field of maltreatment research has focussed on four candidate neurocognitive systems that may embed latent vulnerability, including threat processing, reward processing, emotion regulation and executive functioning (see McCrory *et al.*, 2017 for a recent review). However children exposed to maltreatment are at risk for a wide range of psychiatric conditions, consistent with the concept of multifinality (Cicchetti and Rogosch, 1996); as such, it is likely that a range of other candidate information processing domains are shaped by maltreatment experience. One such domain that has attracted increasing interest in the developmental and psychiatric literature pertains to an individual’s propensity to take risks in the context of potential reward (e.g. Lejuez *et al.*, 2007; Cheng *et al.*, 2012). It is possible that an early adverse environment characterized by unpredictability and/or a paucity of developmentally normative reinforcers may lead to maladaptive calibration of risk-taking propensity. This may result in atypical weighting of risk or reward with serious consequences across development. Reduced risk-taking may lead to a failure to exploit resources within the environment. In contrast, increased risk-taking may lead to greater exposure to adverse outcomes. Only two behavioural experimental studies have investigated risk-taking propensity in maltreated and post-institutionalized children. Guyer *et al.* (2006) used a two-choice decision-making task with reward and punishment contingencies (Wheel of Fortune) and found that children who had experienced maltreatment presenting with depressive disorders tended to select safe over risky choices compared to their peers. A related study using the Balloon Analogue Risk Task (BART), which measures behaviour in the context of increased risk and reward, reported reduced risk-taking in post-institutionalized preadolescent children compared to youth who were internationally adopted early from foster care and non-adopted youth (Loman *et al.*, 2014). These findings were interpreted as reflecting early stressful and unpredictable environments leading to a preference for safe over risky choices (Loman *et al.*, 2014), by decreasing reward sensitivity and increasing punishment sensitivity (Guyer *et al.*, 2006). These studies are consistent with the hypothesis that reduced risk-taking propensity may represent a latent vulnerability factor: similar patterns of altered risk-taking are seen in psychiatric disorders–associated maltreatment such as depression and anxiety disorders (Forbes *et al.*, 2007; Giorgetta *et al.*, 2012).

To date, no prior study has investigated the neurocognitive correlates of risk-taking in individuals exposed to maltreatment. At the neural level, risky decision-making has been related to the interplay of approach and avoidance circuits that have been suggested to form a ‘risk matrix’ (Knutson and Huettel, 2015). In particular, activation of the ventral striatum (VS) has been related to gain anticipation and precedes risky choices (Kuhnen and Knutson, 2005; Canessa *et al.*, 2013), whereas activation of the anterior insula (AI) activation is thought to relate to loss anticipation and precedes safe choices (Paulus *et al.*, 2003; Kuhnen and Knutson, 2005; Canessa *et al.*, 2013).

Alterations in VS and AI function have been implicated in functional neuroimaging studies of childhood maltreatment. For example, adolescents and adults who have experienced childhood maltreatment show attenuated neural activity in VS during reward processing (Dillon *et al.*, 2009; Goff *et al.*, 2013; Hanson *et al.*, 2015; Gerin et al., 2017) and altered neural activity to threat related cues in the amygdala and the AI (McCrory *et al.*, 2011, 2013; Dannlowski *et al.*, 2012; Puetz *et al.*, 2016). Similar atypical functioning of these neurocognitive systems has been implicated in many psychiatric disorders associated with maltreatment such as depression and anxiety disorders (Wolfensberger *et al.*, 2008; Stoy *et al.*, 2012; McCrory and Viding, 2015).

The frontrostriatal brain regions are known to undergo considerable change during development, particularly during adolescence (Steinberg, 2008; Smith *et al.*, 2014). Heightened risk-taking in adolescents compared to adults is thought to relate to greater reward sensitivity of VS (Braams *et al.*, 2014, 2016; van Duijvenvoorde *et al.*, 2016). Adolescents’ risk-taking has also been shown to be particularly sensitive to the social context with the presence of peers associated with heightened VS activation preceding risky decisions (Gardner and Steinberg, 2005; Chein *et al.*, 2011). How peer influence might alter risk-taking propensity in children who have experienced maltreatment remains unclear. Children with a history of maltreatment have been commonly reported to exhibit atypical peer functioning and poorer peer relationships (Bolger *et al.*, 1998), which may alter their susceptibility to peer influence during risk-taking. For example, maltreatment experience has been associated with decreased trust and social motivation (Germine *et al.*, 2015; Pitula *et al.*, 2017), as well as disrupted attachment patterns (e.g. Van Ijzendoorn *et al.*, 1999). Arguably, these responses might be associated with reduced susceptibility to peer influence. On the other hand, children with a history of maltreatment feel more excluded and frustrated after social exclusion (Puetz *et al.*, 2014), and have a greater tendency to associate with deviant peers (e.g. Mandal and Hindin, 2013). These responses may promote susceptibility to peer influence. As such, it remains unclear how altered patterns of peer influence in children with a history of maltreatment would affect susceptibility to peer influence during risk-taking.

The aim of this study was to investigate the neural correlates of risk-taking under varying conditions of peer influence in 10- to 14-year-old children with and without a history of maltreatment. In an automatic version of the BART (Pleskac *et al.*, 2008) during functional magnetic resonance imaging (fMRI), participants pumped up balloons, with each pump increasing the potential reward but also the risk of explosion and thus the loss of the reward. In the original manual version of the BART (Lejuez *et al.*, 2007), the measure of risk-taking propensity (average number of pumps) is biased in the sense that it underestimates risk-taking propensity, as some trials end early (balloon explosion) as the consequence of risk-taking. A more recent version, the automated BART (Pleskac *et al.*, 2008), requires participants to indicate at the beginning of each trial the degree of risk they want to take (how many pumps). This delivers an unbiased estimate of risk-taking propensity (Pleskac *et al.*, 2008). Other advantages of the automated BART include shorter administration time and minimization of motor involvement (Pleskac *et al.*, 2008). To investigate the influence of peers on risk-taking, the participants played the BART under three different conditions: when alone, knowing they were being observed by a peer and having a peer coax them to take risks.

At the behavioural level, we hypothesized that maltreated children would exhibit decreased risk-taking consistent with findings from prior experimental studies of maltreated and post-institutionalized children (Guyer *et al.*, 2006; Loman *et al.*, 2014). At the neural level, we hypothesized that maltreatment experience would be associated with differential modulation of VS and AI by risk level. More specifically, we expected that decreased risk-taking in children who have experienced maltreatment would be associated with altered sensitivity with which the VS and AI activation tracks the level of risk. Specifically, for these children, we predicted reduced modulation of neural activity in the VS by the risk-taking level, and increased modulation of neural activity in the AI by the risk-taking level, based on prior behavioural data. Finally, we explored whether peer influence differentially modulated behaviour and VS and AI functioning in children who had experienced maltreatment and their peers: prior data did not warrant directional hypotheses.

## Materials and methods

### Participants

A total of seventy-five 10–14 year olds were recruited for this study. Forty-one children who had experienced maltreatment (MT group) were recruited from a London Social Services (SS) Department and adoption agencies. Thirty-four non-maltreated children (NMT group) were recruited from schools, youth clubs and via newspaper and Internet advertisement. Exclusion criteria for the NMT group included previous contact with SS with regard to the quality of parental care or maltreatment. Exclusion criteria for all participants included a diagnosis of learning disability, pervasive developmental disorder, neurological abnormalities, standard MRI contraindications (e.g. ferromagnetic implants, past or present neurological disorder) and IQ < 70. Participants across groups were comparable in age, pubertal status, gender, IQ, socio-economic status (level of education of the parents) and ethnicity (see Table 1). Consent was obtained from the child’s legal guardian. Assent to participate in the study was obtained from all children. All procedures in the study were approved by University College London Committee (0895/002).

**Table 1.** Demographic and background information for Maltreated and Nonmaltreated groups

		MT group (*n* = 41)	NMT group (*n* = 34)	
Measure		*Mean (SD)*	*Mean (SD)*	*P*
Age (years)		12.45 (1.49)	12.44 (1.21)	0.89
WASI-IQ^a^		105.17 (12.70)	107.68 (11.62)	0.38
Pubertal Development (PDS)^b^		2.05 (0.70)	1.80 (0.61)	0.11
		*n (%)*	*n (%)*	*P*
Gender (% female)		21 (51)	21 (62)	0.49
Ethnicity (% Caucasian)		28 (67)	20 (59)	0.47
SES^c^		2.82 (1.55)	3.14 (1.12)	0.32
		*Mean (SD)*	*Mean (SD)*	*P*
**CTQ**^d^**(Total)**		37.98 (16.51)	28.72 (4.73)	<0.01
**TSCC**^e^	Depression	47.20 (9.72)	44.29 (8.09)	0.17
	Anxiety	48.56 (12.22)	44.44 (9.65)	0.12

a WASI-IQ, 2-subscale IQ derived from the Wechsler Abbreviated Scales of Intelligence (Wechsler, 1999).

b Self rating of Puberty Development Scale (Petersen et al., 1988).

c Socioeconomic status (SES): Highest level education rated on 6-point scale from 0 = no formal qualifications to 5 = postgraduate qualification.

d Childhood Trauma Questionnaire (Bernstein and Fink, 1998).

e Trauma Symptom Checklist for Children (Briere, 1996).

### Measures

#### Maltreatment experience

For children referred to SS, maltreatment history, including the estimated severity, onset and duration of maltreatment, was provided by the child’s social worker or adoptive parent (on the basis of SS records), using an established maltreatment scale (Kaufman *et al.*, 1994) with an additional rating for intimate partner violence. Severity of each abuse type was rated on a scale from 0 (not present) to 4 (severe). Presence of maltreatment type was rated as follows: neglect *N* = 33; emotional abuse *N* = 40; sexual abuse *N* = 7; physical abuse *N*= 3; exposure to domestic violence *N* = 23. Overall across subtypes, maltreatment was characterized as follows: mean onset in years = 4.14 (s.d. = 4.39), mean duration in years= 5.92 (s.d. = 4.66) and mean severity = 1.54 (s.d. = 0.57) (see Supplementary material for onset, duration and severity by subtype). Additionally, all children completed the self-report Childhood Trauma Questionnaire (Bernstein and Fink, 1998).

#### Cognitive ability

Cognitive ability was assessed using the Wechsler Abbreviated Scales of Intelligence (Wechsler, 1999).

#### Psychiatric symptomatology

To measure symptoms of depression and anxiety, the Trauma Symptom Checklist for Children (TSCC), a self-report measure of affective and trauma-related symptomatology, was administered to all participants (Briere, 1996).

#### Balloon analogue risk task

**Fig. 1.**
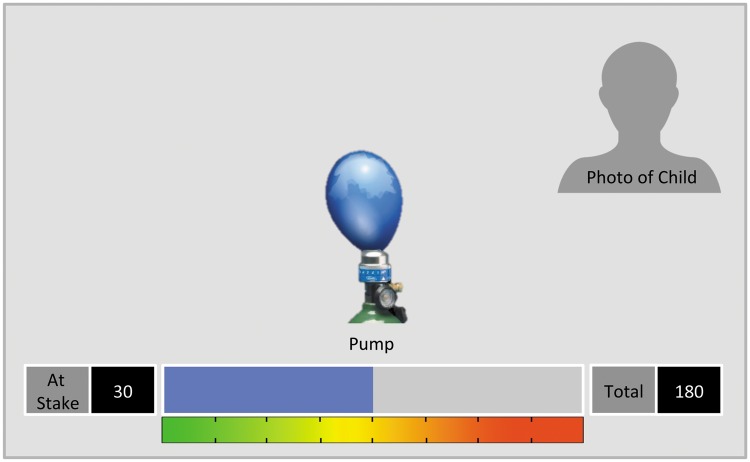
Schematic depiction of the BART during the pumping phase. The photograph of the child was either of themselves (alone condition) or of a peer (observed and peer pressure conditions). We do not have permission to print the actual photographs used in the study.

In this study, we used an automatic version of the BART, as previously described by Pleskac *et al.* (2008), implemented with E-prime v2.0 (Psychology Software Tools, Inc.). During the BART, only one balloon was presented per trial, each having a maximum breaking point of 60 pumps. Participants selected the number of pumps using a button box (corresponding to risk) at the beginning of each trial. A coloured bar at the bottom of the screen indicated the increasing number of pumps. On the bottom left corner, the participants could see how many points were at stake. One pump was worth 10 points. On the bottom right corner, participants could see current earnings (see Figure 1). The decision screen remained visible until the participant made a response. A randomly jittered inter-stimulus interval followed (1.1–2.5 s). Afterwards, the balloon was inflated up to the number of indicated pumps or was interrupted because it exploded. A result screen followed for 1.5–5 s showing the points earned or lost. There were 18 trials per condition (alone, observed and peer pressure).

The BART in this study was adapted to investigate peer influence on risk-taking. Participants were told that there was a group of children who were part of another study at another university, and that the aim of this other study was to investigate whether observing someone playing the BART improved performance. Participants were also told that they would receive real-time feedback about their performance from those children via text messages appearing on the screen in between the trials. Preceding the peer conditions (observed or peer pressure), each child saw a staged Skype video call between the experimenters and another child, asking if the team on the other side was ready. In the alone condition, participants played the BART on their own, receiving general feedback after every 3 trials indicating how many of the 18 trials they had completed; a picture of themselves was present on the upper right corner of the screen. In the observed condition, participants were told another child would be watching them play the BART. This was indicated by a picture of the other child presented on the upper right corner of the screen. Again, for every 3 trials, participants received general feedback indicating how many of the 18 trials they had completed. In the peer pressure condition, participants received feedback from the other child encouraging them to take risks every three trials and the second last trial (e.g. *pump it more next time*). The three BART conditions were administered in three counterbalanced runs of 8 min each. To ensure that all children understood the task, a practice run (not containing any peer element) preceded the scanning session.

#### fMRI data acquisition

Participants were scanned on a 1.5 Tesla Siemens Avanto MRI scanner (Siemens Medical Systems, Erlangen, Germany) using a 32-channel head coil and whole-brain EPI sequence (parameters: voxel size: 3 × 3 × 3 mm, slices per volume: 35; slice thickness: 2 mm; TR: 2975 ms; TE: 50 ms; FoV: 192 mm; gap between slices: 1 mm; flip angle: 90°). A magnetization-prepared rapid gradient-echo sequence (MP-Rage) was used to obtain a high-resolution structural scan (parameters: 176 slices; slice thickness: 1 mm; gap between slices: 0.5 mm; TE: 2730 ms; TR: 3.57 ms; FoV: 256 mm; matrix: 256 × 256 mm; voxel size: 1 × 1 × 1mm). Stimuli were displayed on a front projector and viewed with a mirror mounted on the head coil. All children’s heads were foam padded to minimize head motion.

#### Data analysis

Brain images were analysed using SPM8 (www.fil.ion.ucl.ac.uk/spm/software/spm8), implemented in Matlab 2015 (The MathWorks, 2012). The first three volumes were discarded to allow for T1 equilibrium effects. Pre-processing: Each participant’s scans were realigned within each run and subsequently across all three runs to the first image of run 1. Realigned images were coregistered with the individual anatomical T1-weighted images and subsequently spatially normalized by resampling to a voxel size of 3 × 3 × 3 mm to the standard MNI space (Montreal Neurological Institute). A 6-mm Gaussian filter was applied to smoothen the normalized images and high-pass filtered at 128 Hz.

The pre-processed images were subsequently analysed using the General Linear Model, including the three task regressors, representing: (i) pumping (risk-taking), outcome split by (ii) win outcome (cashout) or (iii) loss outcome (balloon explosion). The risk level in terms of number of pumps was also entered into the model as a linear parametric modulator of the pumping regressor. To reduce movement-related artefacts, we additionally included the six motion parameters and an additional regressor to model images that were corrupted due to head motion >1.5 mm and were replaced by interpolations of adjacent images (<10% of participant’s data for *N* =20 NMT and for *N* = 31 MT, no difference between the groups, *P* = 0.14). For each subject, a contrast of risk for each condition (alone, observed and peer pressure) against the implicit baseline was defined in order to examine the brain activations that covaried with the parametric level of risk. In addition to investigate average brain activity related to risk-taking and feedback processing, contrasts for win and loss outcome as well as pumping were defined for each peer influence condition against the implicit baseline.

A second-level group analysis was conducted using a repeated-measures mixed-effects analysis of variance (ANOVA) by entering the individual statistical parametric maps containing the parameter estimates of the three peer influence conditions as fixed effects and an additional ‘subject factor’ for random effects. This model included the parametric modulators (number of pumps) for the three conditions to investigate brain activation covarying with risk level during the pumping phase. In addition, a second second-level model was conducted that included the main regressors of pumping, win outcome and loss outcome for the three peer influence conditions, to examine average brain activation during risk-taking and outcome (for details and results, see Supplementary material).

In line with our aim to investigate the modulation of AI and VS by the level of risk, region of interest (ROI) analyses were performed using small volume correction (SVC) as implemented in SPM for the AI and the VS, applying family-wise error (FWE) corrections for multiple comparisons. The initial threshold was set to *P* < 0.005 (as for the whole-brain analyses), and an additional extent cluster threshold of *ke* = 5 was applied as an additional precaution to disregard very small activations. AI and VS volumes were functionally defined. The AI volume was based on a parcellation of resting state functional connectivity patterns of the human insula, as provided by Deen *et al.* (2011). The VS volume was based on Martinez *et al.* (2003) who used positron emission tomography to functionally define subdivisions of the striatum. Additional whole-brain analyses were conducted, using Monte-Carlo Simulation (3D ClusterSim; Ward, 2000) correcting for multiple comparisons. Cluster-size-corrected results are reported (voxel-wise *P* < 0.005, *ke* = 75) corresponding *P *=* *0.05, FWE corrected.

Contrast estimates from the peak voxels of clusters where significant group differences emerged were extracted using the MarsBaR Toolbox (Brett *et al.*, 2002) implemented in SPM8 and subsequently correlated with the depression and anxiety scales of the TSCC (Briere, 1996) and maltreatment indices (onset, duration and severity; Kaufman *et al.*, 1994) in SPSS version 21 (IBM Corp. 2012).

## Results

### Behavioural results

To investigate the differential effects of peer influence on risk-taking between MT and NMT groups, a 3 × 2 repeated-measures ANOVA was performed on the mean number of pumps, with peer influence as within-subjects factor (alone, observed and peer pressure) and group as between-subjects factor (MT group *vs* NMT group).

**Fig. 2.**
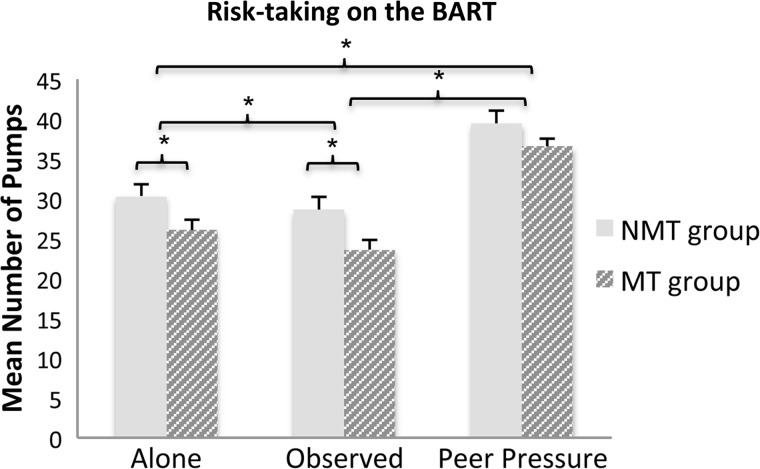
Risk-taking on the BART as measured by mean number of pumps during the different peer influence conditions (error bars: standard error). MT group showed significantly decreased risk-taking across the peer influence conditions.

There were significant main effects of group, *F*(1, 73) = 5.85, *P* < 0.05, η_p_^2^ = 0.07, and condition, *F*(2, 146) = 135.28, *P* < 0.001, η_p_^2^ = 0.65 (see Figure 2). There was no significant group by condition interaction, *F*(2, 146) = 0.94, *P* = 0.39, η_p_^2^ = 0.01, suggesting that both groups were equally susceptible to peer influence. *Post hoc t*-tests showed that risk-taking was significantly reduced in the observed condition compared to the alone condition for both groups [MT group, *t*(40) = −3.41, *P* < 0.01; NMT group, *t*(33) = −2.51, *P* < 0.05]. In addition, risk-taking was significantly increased in the peer pressure condition compared to the observed [MT group, *t*(40) = 10.67, *P* < 0.001; NMT group, *t*(33) = 9.17, *P* < 0.001] and alone condition [MT group, *t*(40) = 8.72, *P* < 0.001; NMT group, *t*(33) = 6.80, *P* < 0.001] for both groups. The MT group engaged in significantly less risk-taking compared to the NMT group in the alone [*t*(73) = 2.15, *P* < 0.05] and the observed [*t*(73) = 2.48, *P* < 0.05] and at trend level in the peer pressure condition [*t*(73) = 1.75, *P* = 0.084].

### fMRI results

In the following, the results of the parametric analysis are presented, investigating modulation of brain activation by level of risk (number of pumps) during the pumping phase (for results of the analyses of average brain activation during pumping and outcome, see Supplementary material).

### *F*-contrast for maltreatment by peer influence interaction: parametric analysis of risk level

In line with the behavioural analysis, we first performed an *F*-contrast to investigate whether there was a significant peer influence (alone, observed and peer pressure) by group (MT group *vs* NMT group) interaction across the whole brain and the AI and VS. Similar to the behavioural findings, there were no significant interaction effects across the whole brain or the AI and VS.

### Main effect of maltreatment: parametric analysis of risk level

**Fig. 3.**
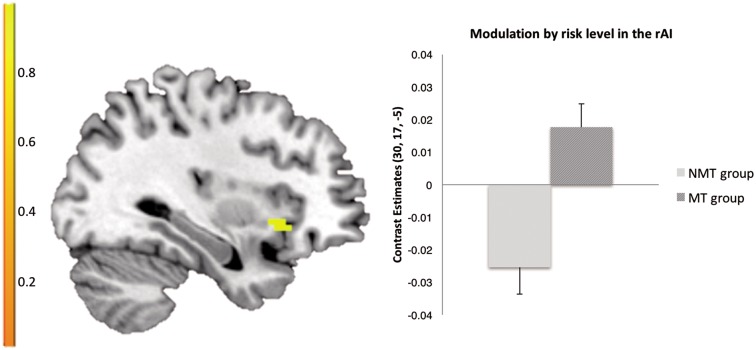
Across all peer influence conditions the MT group exhibited altered modulation of right AI by level of risk (*x* = 30, *y* = 17, *z* = −5) compared to the NMT group (SVC:FWE < 0.05).

To investigate differential modulation by risk level between the two groups, we then performed a *t*-contrast across all peer influence conditions. In our ROI analyses across all peer influence conditions, the MT group exhibited differential modulations of right AI (rAI) activity by level of risk (peak coordinate: *x* = 30, *y* = 17, *z* = −5; *k* = 13, *t* = 3.61, SVC:FWE < 0.05) relative to the NMT group. Whereas the MT group showed a positive rAI modulation by risk level, the NMT group showed a – rAI modulation by risk level (see Figure 3). There was no differential modulation by risk level in the VS between the groups. No whole-brain differences were found between the two groups in our parametric analysis.

### Main effect of peer influence: parametric analysis of risk level

*T*-contrasts were performed incrementally to compare the different levels of peer influence in a systematic way and thus to isolate the unique effects of having another peer observing compared to playing the BART alone (‘observed *vs* alone’) and during peer pressure compared to a peer observing (peer pressure *vs* observed).

**Fig. 4.**
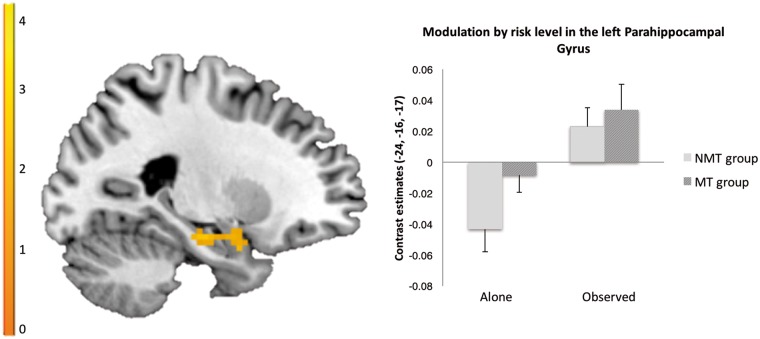
Across the MT and NMT groups whole brain analyses revealed that risk level modulated the left amygdala and left parahippocampal gyrus more strongly in the observed compared to the alone condition (FWE < 0.05).

**Fig. 5.**
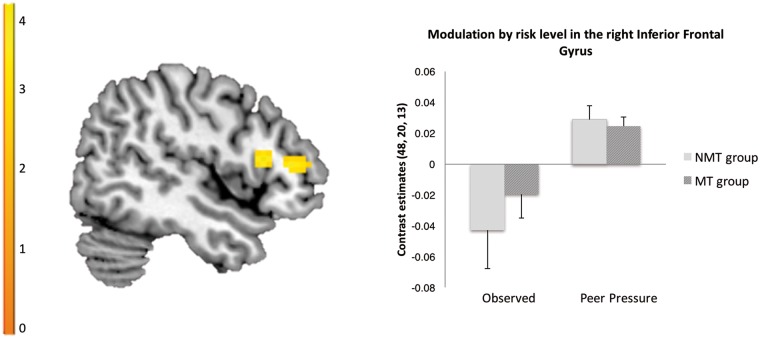
Across the MT and NMT groups whole-brain analyses revealed that risk level modulated the rIFG more strongly in the peer pressure compared to the observed condition (FWE < 0.05).

Across the MT and NMT groups, there was no difference for the contrast ‘observed > alone’ within the AI and VS. Across participants, whole-brain analyses revealed that risk level modulated the left amygdala and left parahippocampal gyrus more strongly in the observed compared to the alone condition (see Table 2 and Figure 4). Across the MT and NMT groups, there was also no difference for the contrast ‘peer pressure > observed’ within the AI and VS. Across participants, whole-brain analyses revealed that risk level modulated the right inferior frontal gyrus (rIFG) more strongly in the peer pressure compared to the observed condition (see Table 2 and Figure 5). No significant interaction effects were found between group and peer influence conditions for the above mentioned contrasts (‘observed > alone’, ‘peer pressure > observed’) within our regions of interest (AI, VS) or across the whole brain.

**Table 2.** Region of interest and whole-brain results of brain activation covarying with risk level (number of pumps)

Brain region	R/L	*x*	*y*	*z*	*ke*	*Z*
Main effect of maltreatment						
*MT group > NMT group*						
Anterior insula^a^	R	30	17	−5	13	3.33
Main effect of peer influence						
*Observed > alone*						
Parahippocampal gyrus	R	12	−37	−2	80	4.39
	R	27	−25	−17		3.10
	R	21	−31	−14		2.69
Parahippocampal gyrus/amygdala	L	−24	−16	−17	90	4.14
	L	−24	2	−23		3.62
	L	−30	−1	−17		3.45
*Peer pressure > observed*						
Inferior frontal gyrus	R	48	20	13	108	4.06
	R	48	35	10		3.81
	R	60	11	16		2.93

a SVC (FWE* *< 0.05).

Abbreviations: R/L, Right/Left; *ke*, cluster extent.

### Relation between risk sensitivity coded in rAI and risk-taking behaviour

To investigate the relation between risk sensitivity coded in the rAI and actual risk-taking behaviour, we extracted the contrast estimates of the significant rAI cluster (based on the main effect of group) and ran a correlation with the risk-taking behaviour.

Risk sensitivity coded in the rAI correlated negatively with risk-taking behaviour over the entire sample, *r* = −0.31, *P* < 0.01. This suggests that heightened risk sensitivity coded in the rAI is associated with decreased risk-taking at the behavioural level.

**Fig. 6.**
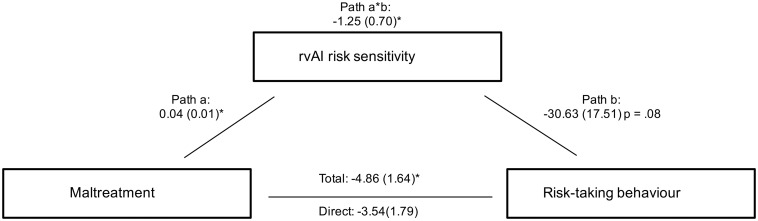
Illustration of the mediation model with risk-taking as the outcome variable, maltreatment (MT group *vs* NMT group) as the independent variable and rAI risk sensitivity as the mediator variable. Values are unstandardized regression coefficients. There was a significant mediation effect of rAI risk sensitivity with respect to the differences in risk-taking between the MT and the NMT groups.

A further mediation analysis was performed to investigate whether differences in rAI risk sensitivity would partially mediate difference in risk-taking between the MT and NMT group. According to Baron and Kenny (1986), three criteria have to be fulfilled for a mediation analysis: (i) the causal variable (in this case group) has to be related to the outcome (in this case risk-taking), (ii) the causal variable has to correlate with the mediator (in this case rAI risk sensitivity) and (iii) the mediator has to have an effect on the outcome variable. Analyses were conducted using bootstrapping procedures recommended for smaller samples and operationalized in an SPSS Macro (Preacher and Hayes, 2008). We used 5000 bootstrap resamples of the data with replacement. Statistical significance with alpha at 0.05 is indicated by the 95% conﬁdence intervals not crossing zero. We found a significant mediation effect of rAI risk sensitivity with respect to the difference in overall risk-taking between the MT and the NMT group (indirect effect = −1.25, SE = 0.70, 95% CI = −2.85 to − 0.04; see Figure 6). In addition, this mediation was total, meaning that individual differences in rAI risk sensitivity accounted fully for the group differences in overall risk-taking.

### *Post hoc* correlations exploring the associations between risk-taking behaviour and maltreatment severity and symptoms of depression and anxiety

We investigated whether risk-taking behaviour in children with a history of maltreatment would relate to maltreatment onset, duration and severity (Kaufman *et al.*, 1994). There was no significant correlation between risk-taking behaviour and maltreatment onset (*r* = 0.06, *P* = 0.69**)** and duration (*r* = −0.10, *P* = 0.55**)**. There was an association at trend level between risk-taking behaviour and maltreatment severity (*r* = −0.28, *P* = 0.07**)**, suggesting that the more severe the maltreatment experience was, the more risk-taking was decreased in children with a history of maltreatment. We also investigated whether risk-taking behaviour in children with a history of maltreatment would relate to self-reported symptoms of anxiety and depression as measured with the TSCC (Briere, 1996). There was no significant association between overall risk-taking and symptoms of anxiety (*r* = −0.19, *P* = 0.23**)** or depression (*r* = −0.27, *P* = 0.09).

### *Post hoc* correlations exploring the associations between risk-sensitivity coded in rAI and maltreatment severity and symptoms of depression and anxiety

We investigated whether risk sensitivity coded in the rAI in children with a history of maltreatment would relate to maltreatment onset, duration and severity (Kaufman *et al.*, 1994). To do so, we extracted the contrast estimates of the significant rAI cluster (based on the main effect of group) and ran correlations with maltreatment onset, duration and severity. There was no significant correlation between risk sensitivity in the rAI and maltreatment onset (*r* = 0.25, *P* = 0.12**)**, duration (*r* = −0.03, *P* = 0.84**)** and severity (*r* = −0.20, *P* = 0.22**)**. We also investigated whether risk sensitivity coded in the rAI in children with a history of maltreatment would relate to self-reported symptoms of anxiety and depression as measured by the TSCC (Briere, 1996). To do so, we extracted the contrast estimates of the significant rAI cluster (based on the main effect of group) and ran correlations with self-reported symptoms of anxiety and depression. Risk-sensitivity coded in the rAI in children who had experienced maltreatment was positively associated with anxiety symptoms (*r* = 0.32, *P* < 0.05**)** but not depression symptoms (*r* = 0.26, *P* = 0.10**)**.

## Discussion

Using fMRI, we investigated risk-taking propensity under varying conditions of peer influence in 10- to 14-year-old children with and without a history of maltreatment. There were three main findings. First, children who had experienced maltreatment engaged in less risk-taking overall during the BART compared to their non-maltreated peers. Second, children who had experienced maltreatment exhibited heightened risk sensitivity in the rAI across peer influence conditions relative to children without a history of maltreatment. Third, experience of maltreatment was not associated with any differential effects of peer influence on risk-taking at the behavioural level, nor at the neural level in our regions of interest (AI, VS) or at the whole-brain level. This suggests that peer influence exerted similar effects during risk-taking irrespective of maltreatment experience.

In line with previous studies (Guyer *et al.*, 2006; Loman *et al.*, 2014), our behavioural findings indicated that children who had experienced maltreatment display decreased risk-taking propensity, indicating a basic preference for safe choices over risky choices. Decreased risk-taking in children with a history of maltreatment potentially reflects heightened loss aversion. In other words, a decreased risk-taking propensity may reflect an adaptation to early adverse environments, in which a ‘safety first’ approach potentially represents the most optimal behavioural strategy. Early adverse environments are characterized by unpredictability and/or a paucity of developmentally normative reinforcers. For many children exposed to such environments, the costs of risk-taking likely outweigh the potential rewards and may be accorded greater salience during decision-making. Albeit speculative, we suggest that decreased risk-taking may serve an adaptive function for these children in early adverse environments, minimizing the likelihood of further experiences of loss and disappointment.

At the neural level, children who had experienced maltreatment showed a differential modulation of the rAI by risk level, irrespective of peer influence, relative to non-maltreated children. Whereas the rAI showed increased activation with risk level in children with a history of maltreatment, the rAI showed decreased activation with risk level in non-maltreated children. Over the entire sample, heightened risk sensitivity in the rAI (greater activation with risk level) was related to reduced risk-taking behaviourally, consistent with the view that AI functioning during risky decision-making is implicated in the inhibition of risky choices, representing a form of loss aversion (Kuhnen and Knutson, 2005; Knutson and Huettel, 2015). Indeed risk sensitivity in the rAI mediated differences in risk-taking between children with and without a history of maltreatment, albeit within a cross-sectional design. This finding needs to be replicated in a longitudinal sample. Abnormal risk sensitivity coded in the rAI in the MT group is also in line with reports of heightened AI activation during threat processing in children who have experienced maltreatment (McCrory *et al.*, 2011). Accumulating evidence thus suggests that maltreatment experience is associated with alteration in neural circuits involved in detecting and anticipating threatening and negative stimuli in the environment. From a developmental perspective, such changes may be hypothesized to disrupt normative risk-taking and reward-seeking behaviour. As we speculate above, greater loss aversion may be adaptive for children in early adverse environments, decreasing potential future losses avoiding repeated disappointments. However, later in life in more normative and stable environments, such heightened loss aversion (indexed by abnormal risk sensitivity in the rAI), might become maladaptive, reducing the degree to which a child successfully explores and exploits the potential for rewards in their new surroundings. Heightened loss aversion and altered rAI functioning, which also characterizes patients with anxiety and depression (Pammi *et al.*, 2015), may thus confer latent vulnerability to future psychiatric disorder for individuals exposed to maltreatment (McCrory and Viding, 2015). Consistent with this notion, our *post hoc* analyses showed that risk sensitivity coded in the rAI was associated with symptoms of anxiety (and at trend level with symptoms of depression) in children who have experienced maltreatment. Interestingly, abnormal AI functioning during learning and decision-making has been found in children with conduct problems and substance abuse (Crowley *et al.*, 2006; White *et al.*, 2013, 2016), who generally tend to exhibit more risk-taking behaviours (Verdejo-García and Pérez-García, 2008; Byrd *et al.*, 2014). Maltreatment has been associated with both conduct problems and substance abuse outcomes (McCrory and Viding, 2015; Puetz and McCrory, 2015) and future research is needed to more fully investigate the neurocognitive risk factors related to these outcomes.

Based on previous findings showing a blunted response of the VS during reward processing in children who have experienced maltreatment (Dillon *et al.*, 2009; Goff *et al.*, 2013; Hanson *et al.*, 2015), we hypothesized reduced modulation by risk level of the VS for children who had experienced maltreatment. However, no differences were observed between the groups in this region. This suggests that decreased risk-taking in children who have experienced maltreatment may primarily be related to increased loss aversion rather than decreased reward seeking, but future tasks that probe punishment avoidance and reward seeking using separate tasks are needed to further elucidate this question.

An additional aim of the study was to investigate whether there were differential effects of peer influence on risk-taking between children with and without a history of maltreatment. Based on previous studies, there were grounds to expect that maltreatment experience may be associated either with greater susceptibility to peer influence (Van Ijzendoorn *et al.*, 1999; Puetz *et al.*, 2014) or reduced susceptibility to peer influence (Mandal and Hindin, 2013; Germine *et al.*, 2015; Pitula *et al.*, 2017). In fact, we found that children who had experienced maltreatment showed normal susceptibility to peer influence during risk-taking. In line with these behavioural findings, no differential effects of peer influence were detected at the neural level across groups. In the observed condition, risk-taking was significantly lower than in the alone condition. Previous studies using the ‘driving task’ in typically developing adolescents reported increased risk-taking in the context of a peer being present (Gardner and Steinberg, 2005; Chein *et al.*, 2011). However, a recent study that also used the BART reported that typically developing adolescents show reduced risk-taking when observed by a peer, consistent with the findings of this study (Kessler *et al.*, 2017). This suggests that the effect of peer presence on risk-taking behaviour is influenced by the specific situational context.

In this study, no differences were found between the observed and the alone conditions in the AI or VS. However, increasing risk level modulated the left and right medial temporal lobe and left amygdala more strongly in the observed condition across groups relative to the alone condition. Heightened amygdala reactivity has been related to loss aversion in previous research (De Martino *et al.*, 2010; Sokol-Hessner *et al.*, 2013), suggesting that the observed pattern of reduced risk-taking in the presence of a peer may in part be associated with increased salience signalling in response to risk level.

During the peer pressure condition, all participants engaged in more risk-taking relative to the observed condition. There was no differential modulation of the AI and VS between these conditions. However, relative to the observed condition, the peer pressure condition modulated the right rIFG more strongly. Heightened risk sensitivity coded in the rIFG in the peer pressure condition, relative to the observed condition, might suggest increased integration of information prior to executing a risky behavioural choice in the peer pressure condition (Dippel and Beste, 2015).

A number of limitations should be noted. First, due to the cross-sectional design, it was not possible to examine the developmental trajectories of altered risk-taking propensity in this sample. Future studies employing longitudinal designs could examine if altered risk sensitivity in rAI predicts future psychopathology in children who have experienced maltreatment, consistent with the suggestion that this may represent a marker of latent vulnerability (McCrory and Viding, 2015). The modest correlations with anxiety and depression symptoms within our non-clinical sample of young adolescents (in the direction expected based on neuroimaging data from clinical samples using risk-taking paradigms) are consistent with this possibility. Second, many clinical studies have reported that maltreatment is associated with an increase of a wide range of complex behaviours understood to reflect ‘risk-taking’ (such as substance misuse or risky sexual behaviours: Bornovalova *et al.*, 2008; Fergusson *et al.*, 2008; Felsher *et al.*, 2010). However, such behavioural outcomes are likely to be underpinned by a diverse set of cognitive processes of which risk-taking propensity, as measured in this study, may only be one. It is not known (for example) whether substance use behaviours associated with childhood maltreatment might represent some form of self-medication behaviour (Puetz and McCrory, 2015) independent of general risk-taking propensity. Future studies should shed light on the likely complex interactions of altered risk-taking propensity and a variety of risk-taking behaviours in adolescents and adults with a history of childhood maltreatment.

In conclusion, the current findings indicate that maltreatment experience is associated with reduced risk-taking and altered risk sensitivity coded in the rAI, but normal susceptibility to peer influence in the context of the BART. Furthermore, altered risk sensitivity in the rAI in children who have experienced maltreatment is related to symptoms of anxiety. Abnormal rAI functioning in children who have experienced maltreatment may therefore disrupt normative risk-taking during development and serve to increase latent vulnerability to future psychopathology. Longitudinal studies are required to test this prediction.

## Supplementary data

Supplementary data are available at *SCAN* online.

## Funding

This work was funded by a grant from the U.K. Economic and Social Research Council (ES/K005723/1) to E.J.M. (PI) and to E.V. who is also a Royal Society Wolfson Research Merit Award holder. We would like to thank the children, parents, carers and social workers who generously participated in this research.

*Conflict of interest*. None declared.

## Supplementary Material

Supplementary Tables
