# Supplementary material for: Risk-taking, peer-influence and child maltreatment: a neurocognitive investigation
Source: Soc Cogn Affect Neurosci. 2017 Oct 23;13(1):124–34. doi: 10.1093/scan/nsx124 (PMC5793726; doi:10.1093/scan/nsx124)
Supplement: Supplementary Tables [file nsx124_supp.pdf]

Supplementary Material

Material and Methods

Measures

Maltreatment experience

For children referred to SS, maltreatment history, including the estimated severity, onset and duration of maltreatment was provided by the child's social worker or adoptive parent (on the basis of SS records), using an established maltreatment scale (Kaufman et al., 1994) with an additional rating for intimate partner violence. Severity of each abuse type was rated on a scale from zero (not present) to four (severe). Presence of maltreatment type was rated as follows: neglect N=33; emotional abuse N=40; sexual abuse N=7; physical abuse N= 3; exposure to domestic violence N=23 (for onset, duration and severity by subtype see Table 1).

Table 1. Abuse subtype severity scores and estimated onset age and duration in years

Abuse Subtype		***Mean***	***SD***
Physical abuse (N=3)			
	Severity	1.00	0.00
	Mean age at onset	4.70	5.09
	Mean duration	5.31	6.52
Neglect (N=33)			
	Severity	3.63	0.74
	Mean age at onset	3.70	4.23
	Mean duration	5.08	4.71
Sexual abuse (N=7)			
	Severity	1.57	0.98
	Mean age at onset	3.44	3.28
	Mean duration	1.43	2.49
Emotional abuse (N=40)			
	Severity	3.10	0.74
	Mean age at onset	3.91	4.19
	Mean duration	5.22	4.74
Domestic Violence (N=23)			
	Severity	2.52	1.20
	Mean age at onset	4.20	4.63
	Mean duration	3.56	3.40

Balloon Analogue Risk Task (BART)

‘In the peer pressure condition participants received a message from the other peer encouraging them to take more risks. These messages appeared in screen after every 3 trials and before the last trial. The preset messages used for this study can be seen in Table 2. The feedback messages were preset and appeared independently of the number of pumps the participants used during the trials.

Table 2. Feedback messages during the Peer Pressure condition

After 3 trials	‘hey! try the red part’
After 6 trials	‘pump it more next time’
After 9 trials	‘more points if you pump more☺’
After 12 trials	‘pump ALL the way’
After 15 trials	‘cmon pump more this time’
After 17 trials	‘Last chance! Go 4 it!!

fMRI Analysis

The preprocessed images were subsequently analysed using the General Linear Model, including the three task regressors, representing: a) pumping (risk-taking), outcome split by b) win outcome (cashout) or c) loss outcome (balloon explosion). The risk level in terms of number of pumps was also entered into the model as a linear parametric modulator of the pumping regressor. To reduce movement-related artefacts, we additionally included the six motion parameters and an additional regressor to model images that were corrupted due to head motion >1.5 mm and were replaced by interpolations of adjacent images (<10% of participant’s data for N =20 NMT and for N

= 31 MT, no difference between the groups, p = .14). To investigate average brain activity related to risk-taking and feedback processing, contrasts for win and loss outcome, as well as pumping were defined for each peer influence condition against the implicit baseline.

A second-level group analysis was conducted using a repeated measures mixed-effects ANOVA by entering the individual statistical parametric maps containing the parameter estimates of the 3 peer influence conditions as fixed effects and an additional ‘subject factor’ for random effects. This model included the the main regressors of pumping, win outcome and loss outcome for the 3 peer influence conditions, to examine average brain activation during risk-taking and outcome.

Whole brain analyses were conducted, using Monte-Carlo Simulation (3D ClusterSim; Ward, 2000) correcting for multiple comparisons. Cluster-size corrected results are reported (voxel-wise $p<0.005$, $ke=75$) corresponding $p=0.05$, family-wise error (FWE) corrected.

fMRI Results

Risk-taking (pumping)

Main effect of pumping

Across groups and peer influence conditions risk-taking significantly activated among others left and right anterior insula, dorsolateral prefrontal cortex, inferior parietal lobule and middle cingulate cortex and striatum (see Table 3).

Main effect of peer influence

T-contrasts were performed incrementally to compare the different levels of peer influence. This was done to isolate the unique effects of having another peer observing compared to playing the BART alone ('observed vs. alone') and during peer pressure compared to a peer observing (peer pressure vs. observed).

Across the maltreated (MT) and non-maltreated (NMT) children risk-taking during the observed condition relative to the alone condition activated more strongly left lentiform nucleus, left middle frontal gyrus, right postcentral gyrus and right middle frontal gyrus (see Table 3). Across the MT and NMT groups risk-taking during the peer pressure condition relative to the observed condition activated more strongly right lingual gyrus, right cingulate gyrus, right superior frontal gyrus, right cerebellum, left precentral gyrus (see Table 3).

Main effect of maltreatment

Across the peer influence conditions the NMT group showed greater activation in the left anterior insula during risk-taking compared to the MT group (see Table 3).

Maltreatment by peer influence interactions

The NMT group showed significantly stronger activation relative to the MT group for the 'observed>alone' contrast in the left superior temporal gyrus and superior frontal gyrus (see Table 3). The MT group showed significantly stronger activation relative to

the NMT group for the ‘peer pressure>observed’ contrast in the right medial frontal gyrus (see Table 3).

Table 3: Whole-brain results of average brain activation during risk-taking (pumping)

**Brain region**	**R/L**	**x**	**y**	**z**	***ke***	***Z***
**Main effect of risk-taking (pumping across conditions)**						
Anterior Insula	R	33	17	7	18105	>6
	R	54	-31	49		>6
	R	39	14	1		>6
**Main effect of peer influence (across groups)**						
Observed>alone						
Lentiform Nucleus	L	-18	-4	16	90	4.25
	L	-15	8	13		3.49
Middle Frontal Gyrus	L	-33	50	4	178	4.21
	L	-15	8	13		4
	L	-18	59	4		2.99
Postcentral Gyrus	R	54	-34	52	84	3.86
	R	45	-34	52		3.7
	R	42	-43	52		3.08
Middle Frontal Gyrus	R	36	8	58	112	3.68
	R	33	-1	58		3.54
	R	42	20	52		3.52
Peer pressure>observed						
Lingual Gyrus	R	12	-88	1	730	5.85
	R	24	-73	-5		5.15
	R	27	-73	10		4.9
Cingulate Gyrus	R	24	-40	34	123	4.55
	R	18	2	28		4.35
	R	27	-13	28		4.15
Superior Frontal Gyrus	R	24	65	1	309	4.55
	R	48	38	22		4.49
	R	33	59	16		4.44
Cerebellum	R	6	-49	4	102	4.39
	L	-6	-43	-2		3.51
	R	15	-40	-11		2.85
Precentral Gyrus	L	-18	-22	61	104	4.19
	L	-24	-31	61		4.11

	L	-9	-16	61		3.6
**Main effect of group (across conditions)**						
NMT>MT						
Anterior Insula	L	-36	8	-2	215	3.78
	L	-54	5	1		3.77
	L	-39	-1	-2		3.56
MT>NMT						
		-	-	-	-	-
**Group by peer influence interaction (**						
Observed>alone, NMT>MT						
Superior Temporal Gyrus	L	-57	-55	28	187	4.83
	L	-48	-64	31		3.82
	L	-39	-73	37		3.81
Superior Frontal Gyrus	R	15	35	52	119	4.05
		0	50	43		3.54
	L	-6	38	52		3.43
Observed>alone, MT>NMT						
		-	-	-	-	-
Peer pressure>observed, NMT>MT						
		-	-	-	-	-
Peer pressure>observed, MT>NMT						
Medial Frontal Gyrus	R	3	53	34	88	4.44
		6	62	25		3.5
		12	47	46		2.93

Note. Abbreviations: R/L, right/left; ke, cluster extent

Win outcome

Main effect of win outcome

Across groups and peer influence conditions relative to the loss outcome the win outcome significantly activated left and right caudate (see Table 4).

Main effect of peer influence

T-contrasts were performed incrementally to compare the different levels of peer influence. This was done to isolate the unique effects of having another peer observing compared to playing the BART alone ('observed vs. alone') and during peer pressure compared to a peer observing (peer pressure vs. observed).

Across the MT and NMT groups the win outcome during the observed condition relative to the alone condition activated more strongly left caudate and the posterior cingulate (see Table 4). There were no significant differences in brain activation for the win outcome in the peer pressure condition relative to the observed condition.

Main effect of maltreatment

Across the peer influence conditions the NMT group showed greater activation in the anterior cingulate compared to the MT group for 'win > loss' (see Table 4).

Table 4: Whole-brain results of average brain activation during win outcome (win>loss)

Brain region	R/L	x	y	z	*ke*	*Z*
**Main effect of win outcome win>loss (across conditions)**						
Caudate	R	9	17	7	4675	>8
	L	-6	-34	16		7.4
	L	-27	-52	19		7.19
**Main effect of peer influence on win>loss (across groups)**						
Observed>alone						
Caudate	L	-6	8	-5	91	4.2
	L	-12	-7	-8		3.88
	L	-15	2	-11		3.15
Posterior Cingulate	R	6	-49	13	735	4.11
	R	27	-88	25		4.1
	L	-12	-55	7		4.02
Peer pressure>observed						
		-	-	-	-	-
**Main effect of group, win>loss (across conditions)**						
NMT>MT						
Anterior Cingulate	R	12	29	-2	88	3.97
	R	3	23	-5		3.64
	L	-3	11	1		3.02
MT>NMT						
		-	-	-	-	-

Note. Abbreviations: R/L, right/left; ke, cluster extent

Loss outcome

Main effect of loss outcome

Across groups and peer influence conditions relative to the win outcome the loss outcome significantly activated right cerebellum, left and right occipital lobe, left and right inferior frontal gyrus, right supramarginal gyrus, right middle temporal gyrus and left medial frontal gyrus (see Table 5).

Main effect of peer influence

Across the MT and NMT groups there was no sigificant differences in brain activation for the ‘observed > alone’ or for ‘peer pressure > observed’ (see Table 5).

Main effect of maltreatment

Across the peer influence conditions the MT group showed greater activation in the anterior cingulate compared to the NMT group, for ‘loss > win’ (see Table 5).

Table 5: Whole-brain results of average brain activation during loss outcome (loss>win)

Brain region	R/L	x	y	z	*ke*	*Z*
**Main effect of loss outcome loss>win (across conditions)**						
Cerebellum	R	30	-58	-11	315	>8
	R	27	-46	-14		7.19
	R	48	-61	-11		5.77
Occipital Lobe	L	-24	-64	-8	564	>8
	L	-3	-82	-2		7.02
	L	-30	-88	10		4.68
Inferior Frontal Gyrus	L	-36	20	-14	352	7.25
	L	-30	5	-17		4.59
	L	-36	-10	-8		3.83
Middle Temporal Gyrus	R	48	-31	-2	172	6.41
Inferior Frontal Gyrus	R	33	20	-17	346	6.38
	R	45	23	-8		5.79
		30	5	-17		4.63
Occipital Lobe	R	39	-82	7	152	5.69
Medial Frontal Gyrus	L	-6	53	16	287	4.41
	L	-3	26	19		3.85
	R	6	35	19		3.73
Supramarginal Gyrus	R	60	-46	28	101	4.29
	R	66	-37	28		3.91
**Main effect of peer influence on loss>win (across groups)**						
Observed>alone						
		-	-	-	-	-
Peer pressure>observed						
		-	-	-	-	-
**Main effect of group, loss>win (across conditions)**						
NMT>MT						
		-	-	-	-	-
MT>NMT						
Anterior Cingulate	R	12	29	-2	88	3.97
	R	3	23	-5		3.64
	L	-3	11	1		3.02

Note. Abbreviations: R/L, right/left; ke, cluster extent

References

Kaufman, J., Jones, B., Stieglitz, E., Vitulano, L., Mannarino, A. P. (1994). The use of multiple informants to assess children's maltreatment experiences. *Journal of Family Violence*, **9**, 227–48.
